# The Role of Perceived Social Support in the Association Between Stressful Life Events and Suicidal Behavior

**DOI:** 10.3389/fpsyt.2021.699682

**Published:** 2021-09-10

**Authors:** Balpreet Panesar, Tea Rosic, Myanca Rodrigues, Nitika Sanger, Natasha Baptist-Mohseni, Alannah Hillmer, Caroul Chawar, Alessia D'Elia, Luciano Minuzzi, Lehana Thabane, Zainab Samaan

**Affiliations:** ^1^Neuroscience Graduate Program, McMaster University, Hamilton, ON, Canada; ^2^Department of Psychiatry and Behavioral Neurosciences, McMaster University, Hamilton, ON, Canada; ^3^Health Research Methodology Graduate Program, McMaster University, Hamilton, ON, Canada; ^4^Medical Science Graduate Program, McMaster University, Hamilton, ON, Canada; ^5^Department of Psychology, York University, Toronto, ON, Canada; ^6^Department of Health Research Methods, Evidence and Impact, McMaster University, Hamilton, ON, Canada; ^7^Centre for Evaluation of Medicines, Programs for Assessment of Technology in Health (PATH) Research Institute, McMaster University, Hamilton, ON, Canada

**Keywords:** suicide, mediation, moderation, stressful life events, social support

## Abstract

**Background:** Suicide is a serious public health concern for which there have been well-established protective and risk factors reported in literature. There is a lack of evidence on the indirect effects of other variables on these factors. Specifically, the association between stressful life events and suicidal behavior may be affected by perceived social support, but its role in this association is largely uninvestigated.

**Objectives:** Thus, this paper aims to explore the role of perceived social support in the association between stressful life events and suicidal behavior. Perceived social support will be explored as a mediator and as a moderator in this association.

**Methods:** Data were obtained from the Determinants of Suicidal Behavior Conventional and Emergent Risk (DISCOVER), a study conducted to identify risk factors of suicidal behavior. The study participants are individuals with suicide attempts admitted to hospital. Participants (*n* = 343) were recruited from hospital setting. Suicidal behavior was measured using two outcomes (1) the occurrence of a suicide attempt (2) level of suicide intent as measured by the Pierce Suicide Intent Scale. Perceived social support was measured using the Sarason Social Support Questionnaire.

**Results:** Stressful life events were significantly associated with suicide attempts (OR 1.440, 95% CI 1.440, 1.682, *p* < 0.001) and perceived social support (B −0.785, 95% CI −1.501, −0.068, *p* = 0.032). There was no significant mediation effect by perceived social support in the association between stressful life events and suicide attempts (Sobel's test statistic 1.64, *p* = 0.100). Perceived social support did not moderate the relationship between stressful life events and suicide attempts [(OR 1.007, 95% CI 0.987, 1.027, *p* = 0.514] or the relationship between stressful life events and level of suicidal intent (B −0.043, 95% CI −0.132, 0.046, *p* = 0.343).

**Conclusion:** Stressful life events are associated with increased risk of suicide attempts. The study also identified an inverse relationship between stressful life events and perceived social support. These associations were independent of perceived social support. This study highlights the effects of stressful life events on suicide risk is not affected by perceived social support, requiring further investigation into measures to reduce the impact of social stressors on people with risk of suicide.

## Background

Suicide and suicidal behavior are serious social and health concerns. Every year, about 800,000 people worldwide die by suicide, and it is one of the leading causes of death for 15–29 years old (1). Researchers have identified a multitude of risk and protective factors for suicide in recent years. Factors such as depression (2–6), family history of suicide (7–9), and stressful life events (10–14) have been found to have direct relationships with suicidal behavior. It is important to elaborate on these well-established direct effects to uncover and clarify causal links through which these effects may occur, and to ultimately inform suicide prevention and intervention efforts with specific, defined pathways rather than broad associations. As such, a growing area of research interest is the examination of the indirect effects of such factors on suicidal behavior, and interactions between different risk or protective factors (15, 16). Recently, studies have attempted to clarify indirect effects of well-established risk factors for suicide; for example, studies looking at depression and suicide that suggest the inclusion of variables such as life satisfaction and psychiatric status to induce the causal pathway between depression and suicide (17, 18). The recent evidence for the influence of different factors on the well-established association between depression and suicide presents reason to clarify the links between other known associations relating to suicidal behavior. One such established association is between stressful life events and suicidal behavior, for which a direct pathway has been consistently reported (10–14).

Stressful life events are defined as unexpected and undesired life events that often have the capacity to influence health and well-being (19). The association between stressful life events and suicidal behavior has been extensively studied, but there is limited evidence on indirect factors that moderate or mediate the relationship between the two variables. Perceived social support is defined as the individual's subjective understanding of the level of social support that can be provided by members of their social network (20). Current literature has shown moderating effects of perceived social support in the association between stressful life events and depression (21, 22), stressful life events and hopelessness (23, 24), and stressful life events and subjective well-being (25). There is also evidence for perceived social support as a moderator in the association between stressful life events (i.e., traumatic events, discrimination, medical diagnosis) and suicidal behavior in specific populations such as South African adolescents and transgender samples (26–28). There is limited evidence for perceived social support as a mediator (29) in the association between stressful life events and suicidal behavior but there is reason to suggest that perceived social support and stressful life events are correlated. Specifically, there are studies that show there is deterioration of perceived social support after the occurrence of a stressful life event (30, 31), and there has been a recent study in adolescents that established perceived social support as a mediator in the association between stressful life events and suicidal ideation and suicide attempts (32). Furthermore, a study looking at relationship between stress and health suggests that the stress-health connection is influenced by perceived social support, and can only be appropriately understood after the consideration of moderating and mediating factors (19). Thus, although there is limited evidence of perceived social support as either a moderator or mediator in the association between stressful life events and suicide, there are indications in literature that perceived social support is involved in the association between stressful life events and suicidal behavior. Furthermore, stressful life events are risk factors that are difficult to address with suicide prevention strategies as they can be unpredictable events (33, 34). In contrast, social support is a modifiable factor that is likely able to be influenced by suicide related prevention and intervention strategies, and as such, more investigation is needed to determine how perceived social support fits into the association between stressful life events and suicidal behavior.

This study will expand on current literature by investigating the role of perceived social support in the association between stressful life events and suicidal behavior in patients with suicide attempts admitted to psychiatric hospital. The limited evidence on the role of perceived social support in the association, combined with the established direct associations between stressful life events and suicidal behavior (10–14), perceived social support and suicidal behavior (35–37), and adequate evidence for stressful life events and perceived social support (30–32) provide further reason to clarify the associations between these three variables. Thus, this study will explore the role of perceived social support in the association between stressful life events and suicidal behavior by assessing its role as a mediator or moderator. The findings from this study will add to current literature that is defining the indirect effects of variables on well-established relationships between protective and risk factors for suicide and suicidal behavior. The goal of this research is to identify an actionable modifiable factor such as social support to reduce the risk of suicide.

## Objectives

The objectives of this study are to explore the role of perceived social support in the association between stressful life events and suicidal behavior by investigating if:

1 the association between stressful life events, reported within the last year, and the occurrence of suicide attempts is mediated by the self-reported level of perceived social support,
2 the association between stressful life events, reported within the last year, and the occurrence of suicide attempts is moderated by the self-reported level of perceived social support,
3 the association between stressful life events, reported within the last year, and level of suicide intent is mediated by the self-reported level of perceived social support,
4 the association between stressful life events, reported within the last year, and level of suicide intent is moderated by the self-reported level of perceived social support.

## Methods

### Reporting

The study is reporting according to the Strengthening of the Reporting of Observational Studies in Epidemiology (STROBE) statement for cross-sectional studies (38).

### Setting and Study Design

The data from this study were obtained from the case-control study titled Determinants of Suicidal Behavior Conventional and Emergent Risk (DISCOVER), which was conducted to identify risk factors of suicidal behavior (39, 40). The study was approved by the Hamilton Health Sciences (#10-661) and St. Joseph's Healthcare (#11-3479) Research Ethics Boards. The study design for this paper is cross-sectional as data were taken from questionnaires administered at one time point. Study recruitment was completed from two city hospitals including Hamilton General Hospital, and St. Joseph's Healthcare, Hamilton where psychiatric inpatient services provided.

### Participants

The inclusion criteria for the cases (individuals with suicide attempt) in this study were adult (18 years or older) participants that were admitted to hospital (the psychiatric or general hospitals), had a recent or past suicide attempt. The inclusion criteria for the controls in this study were psychiatric inpatients admitted at the same time as the cases with no history of suicide attempts and non-psychiatric inpatients without a history of suicide attempts. All study participants provided written informed consent and were interviewed face-to-face to conduct study related procedures. Participants were recruited from February 2011 to December 2014.

### Variables

The socio-demographic characteristics included in this study were age, sex, ethnicity, marital status, current employment, and satisfaction with social support. Marital status was operationalized as those with a partner (currently married, common law, living with a partner) and those without a partner (never married, widowed, separated, divorced). Satisfaction with social support was measured using the Sarason Social Support Questionnaire (SSQ) short form, which asks participants how satisfied they were with the social support they were receiving on a six-point rating scale ranging from “very dissatisfied” to “very satisfied.” (41). It asks participants to list up to 10 people who they can count on to be dependable, who can help them relax when under pressure, who accept their worst and best points, who really care about them, who can help them feel better, and who can console them. A social support score is generated by means of the number of people the participant has listed for each of the 6 items that were asked. The higher the number of the satisfaction with social support score, the greater the satisfaction of the participant. The satisfaction with social support score is generated by the means of the satisfaction ratings for all 6 items included in the SSQ (41). The Cronbach alpha for inter-reliability for this questionnaire is 0.97 (41).

Information about any stressful life events the participants experienced in the last year was also collected. Stressful life events included the dichotomous variables of marital separation, loss of job/retirement, loss of crop/business failure, violence, major intra-family conflict, major personal injury or illness, death/major illness of a close family member, death of a spouse, and other. These dichotomous variables were coded, where no occurrence of the stressful life event was coded as zero and the occurrence of a stressful life event was coded as one. These coded variables were then added to create a continuous stressful life events score.

Suicidal behavior was assessed using the occurrence of a suicide attempt and the level of suicidal intent. The occurrence of a suicide attempt was determined by self-reported suicide history data, which was then confirmed through access to medical records (39). The questionnaire used in the DISCOVER study to evaluate the level of suicidal intent was the Pierce Suicide Intent Scale (42). The Pierce Suicide Intent Scale is comprised of 12 questions that generate an output score of 0–22. A score of 0–3 on the questionnaire indicates low intent, 4–10 indicates medium intent, and 10+ indicates high intent (42). The Cronbach alpha value for the Pierce Suicide Intent Scale is 0.77 (43).

### Statistical Analysis

Version 26 of SPSS (44) was used to conduct all descriptive, mediation, moderation, and interaction analyses. Descriptive statistics were used to summarize the demographic data of the study population. Continuous variables are presented as means with standard deviations, while dichotomous variables are summarized using percentages.

Our first objective was to explore whether the association between stressful life events and suicide attempts is mediated by level of perceived social support. Our mediation analysis was conducted in three steps, as follows:

1 First, we assessed the association between stressful life events score (independent variable) and suicide attempt (dependent variable) using a logistic regression model with age and sex included as covariates.
2 Second, we assessed the association between stressful life events score (independent variable) and social support score (mediator) using a linear regression model with age and sex included as covariates.
3 Third, we constructed a final logistic regression model with suicide attempt included as the dependent variable, and both social support score and stressful life events score as independent variables, adjusting also for age and sex. If social support exerted a mediation effect, the association between stressful life events and suicide attempt would diminish as compared to that detected in step one of our analysis. We then applied Sobel's test to assess the statistical significance of the mediation effect using an online calculator (45).

Our second objective was to explore whether the association between stressful life events and suicide attempts is moderated by level of perceived social support. Our moderation analysis was conducted as follows: we created a continuous interaction term using the product of the stressful life events score and the social support score and tested this interaction in a logistic regression model using suicide attempt as the dependent variable. Our model was adjusted for age and sex, as well as the social support score, and the stressful life events score.

Our third objective was to explore whether the association between stressful life events and level of suicide intent is mediated by level of perceived social support. This mediation analysis was conducted using the same steps as described earlier:

1 First, we assessed the association between stressful life events score (independent variable) and level of suicide intent (dependent variable) using a linear regression model with age and sex included as covariates.
2 Second, we assessed the association between stressful life events score (independent variable) and social support score (mediator) using a linear regression model with age and sex included as covariates.
3 Third, we constructed a final linear regression model with level of suicide intent included as the dependent variable, and both social support score and stressful life events score as independent variables, adjusting for age and sex. If social support exerted a mediation effect, the association between stressful life events and level of suicide would diminish as compared that detected in to step one of our analysis. Sobel's test was used to assess the statistical significance of the mediation effect (45).

Our fourth objective was to explore whether the association between stressful life events and level of suicide intent is moderated by the self-reported level of perceived social support. We created a continuous interaction term using the product of the stressful life events score and the social support score and tested this interaction in a linear regression model using level of suicidal intent as the dependent variable. Our model was adjusted for age and sex, as well as the social support score, and the stressful life events score.

For logistic regression analyses, we report odds ratios (OR) with 95% confidence interval (CI), and p-values. For linear regressions, we report unstandardized Beta-coefficients (*B*) with 95% CI, and p-values. For all analyses the alpha level of significance was set to *a* = 0.05. We assessed for multicollinearity using the variance inflation factor (VIF), where a VIF less than two was considered to be an acceptable cut-off.

## Results

### Demographic Characteristics

**Figure 1:**
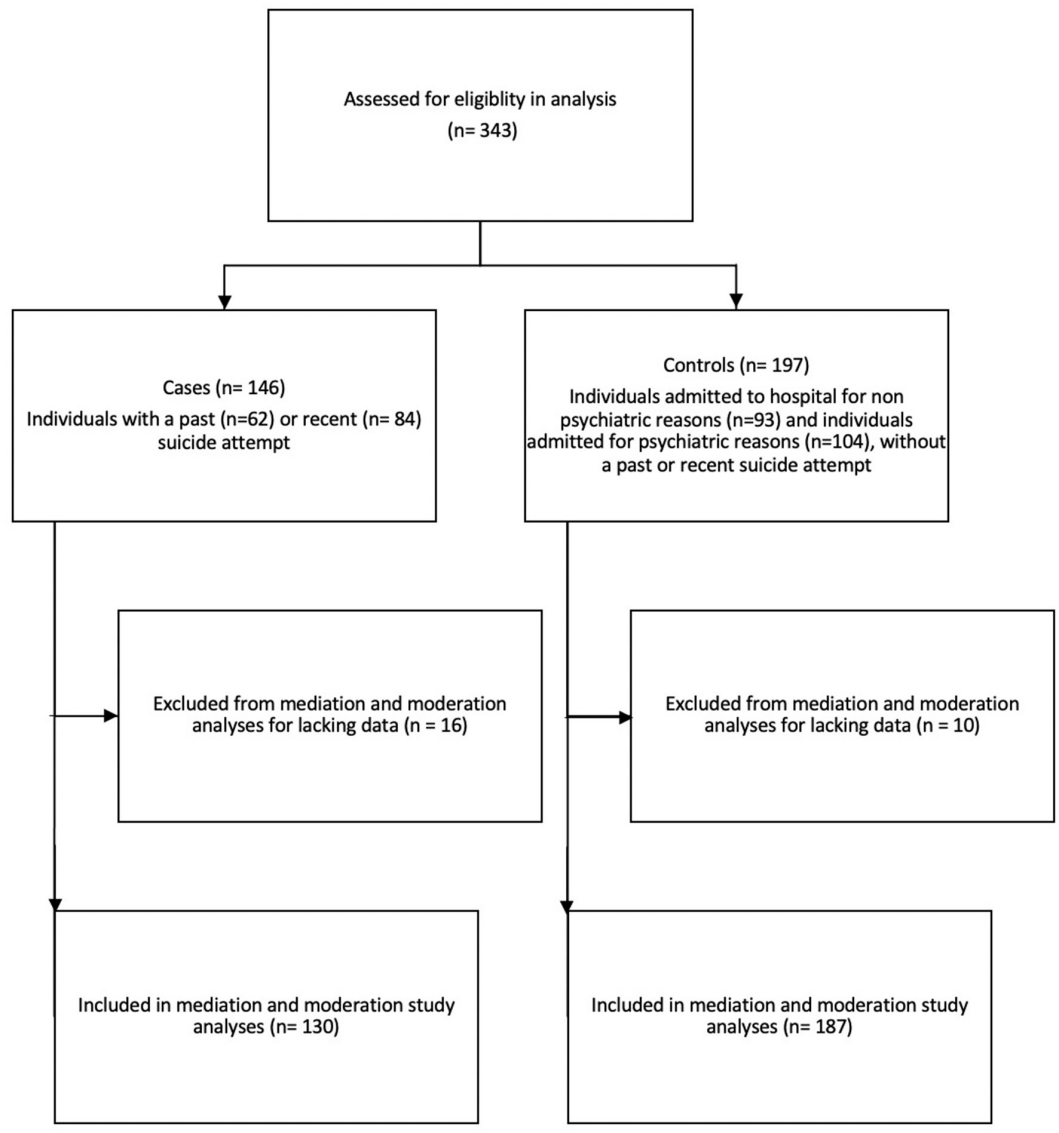
Study flow diagram.

Altogether, 343 individuals included in this study, 146 of whom were cases with a past or recent suicide attempt, and 197 of whom were controls (Figure 1). There were more women than men (women cases 55.5%, women control 49.7%) in the cases compared to the controls, and the cases were more likely to be without a partner (cases without partner 73.1%, controls without partner 59.4%). There was a larger proportion of individuals with European ethnicity seen in the cases group (92.5%) compared to the control group (72.8%). The mean social support score was 9.11 SD 8.88) compared to controls (12.5 SD 10.8), in contrast to the stressful life events score which was higher in cases (11.4 SD 1.53) compared to controls (10.5 SD 1.52). One of the highest reported stressful life events was “other stressful life event” where 44.6% of cases reported having other and 32.6% of controls reported having other stressors. Examples of these “other” stressors are “inability to find work,” “financial stress,” “having a baby,” and “housing issues.” The study participants' characteristics are provided in Table 1.

**Table 1:** Demographic characteristics of participants with and without a history of suicide attempts.

**Demographic variable**	**Total** **(** ***n*** **= 343)**	**Participants with a history of suicide attempts** **(*n* = 146)**	**Participants without a history of suicide attempts** **(*n* =197)**
Sex; % male	47.8	44.5	50.3
Mean Age in years (SD)	45.5 (15.4)	45.2 (14.7)	45.7 (15.9)
Ethnicity; % European^a^	81.2	92.5	72.8
Marital status; % with a partner^a^	34.9	26.9	40.6
Employment status; % currently employed^b^	35.6	23.3	44.7
Mean social support score (SD)	11.1 (10.2)	9.11 (8.88)	12.5 (10.8)
Mean satisfaction with social support score (SD)	6.9 (7.05)	7.58 (8.16)	6.36 (6.08)
Mean stressful life events score (SD)^c^	10.9 (1.6)	11.4 (1.53)	10.5 (1.52)
Marital separation^e^; % yes	9.4	12.2	7.5
Loss of job/retirement^e^; % yes	17.0	19.8	15.0
Loss of crop/business failure^e^; % yes	3.5	4.6	2.7
Violence^e^; % yes	11.9	16.0	9.1
Major intra-family conflict^e^; % yes	33.6	46.6	24.6
Major personal injury/illness^e^; % yes	37.1	52.7	26.2
Death or major illness of a family member^e^; % yes	34.3	38.9	31.0
Death of a spouse^e^; % yes	0.9	0.8	1.1
Other stressful life event^c^; % yes	37.5	44.6	32.6
Mean suicide intent score (SD)	-	14.2 (4.73)^d^	-

a *A total of 341 participants had data for this variable*.

b *A total of 342 participants had data for this variable*.

c *A total of 317 participants had data for this variable*.

d *A total of 115 participants had data for this variable*.

e *A total of 318 participants had data for this variable*.

### Perceived Social Support as a Mediator of the Association Between Stressful Life Events and Suicide Attempt

We found a significant association between stressful life events and suicide attempts, such that for each additional stressful life event, the odds of having a suicide attempt were 1.440 times greater (OR 1.440, 95% CI 1.440, 1.682, *p* < 0.001; Table 2). We also found a significant association between stressful life events and social support; each additional stressful life event was associated with a decrease in social support score (B −0.785, 95% CI −1.501, −0.068, *p* = 0.032). Our mediation analysis, which included both perceived social support and stressful life events as independent variables, revealed significant associations between both stressful life events (OR 1.414, 95% CI 1.209, 1.655, *p* = 0.001) and perceived social support (OR 0.967, 95% CI 0.942, 0.993, *p* = 0.013) and suicide attempt. The OR for stressful life events decreased from 1.440 in the first analysis to 1.414 in the mediation analysis, however, this effect did not reach the threshold of statistical significance when the Sobel's test was conducted (test statistic 1.64, *p* = 0.100). Details on the models used to conduct mediation analyses can be found in Table 2.

**Table 2:** Logistic and linear regression results from mediation and moderation analyses with suicide attempts as the outcome^a^.

	**Predictors**	**Outcome**	**OR**	**95% CI**	***p***
Model 1	Age	Suicide attempts	1.001	0.985, 1.016	0.928
	Female		1.298	0.813, 2.074	0.275
	SLEs^c^		1.440	1.440, 1.682	<0.001^*^
Model 2^b^	Age	Social support	−0.004	−0.078, 0.069	0.906
	Female		−0.454	−2.694, 1.787	0.691
	SLEs^c^		−0.785	−1.501, −0.068	0.032^*^
Model 3	Ag	Suicide attempts	1.001	0.985, 1.016	0.948
	Female		1.284	0.800, 2.061	0.301
	SLEs^c^		1.414	1.209, 1.655	<0.001^*^
	Perceived social support		0.967	0.942, 0.993	0.013^*^
Model 4	Age	Suicide attempts	1.000	0.985, 1.016	0.954
	Female		1.297	0.807, 2.085	0.282
	Perceived social support		0.901	0.726, 1.118	0.344
	SLEs^c^		1.324	1.031, 1.701	0.028^*^
	SLEs^c^ × Perceived social support		1.007	0.987, 1.027	0.514
**Sobel's test**			**Sobel's test statistic**	**Standard error**	***p***
			1.64	0.016	0.100

a *Models 1 tests the independent association between SLEs and suicide attempts. Model 2 tests the independent association between SLEs and perceived social support. Model 3 represents the mediation analysis. Model 4 represents the moderation analysis*.

b *Unstandardized B values are reported for this linear regression*.

c *Stressful life events (SLEs)*.

* *Statistically significant at the 0.05 level*.

### Perceived Social Support as a Moderator of the Association Between Stressful Life Events and Suicide Attempt

Perceived social support was not a moderator of the association between stressful life events and suicide attempt, as evidenced by a non-significant interaction effect between perceived social support and stressful life events (OR 1.007, 95% CI 0.987, 1.027, *p* = 0.514; Table 2).

### Perceived Social Support as a Mediator of the Association Between Stressful Life Events and Level of Suicide Intent

We did not find a significant association between stressful life events and level of suicidal intent reported by participants (B 0.040, 95% CI −0.571, 0.652, *p* = 0.896; Table 3). No mediation effect of perceived social support could be detected (Table 3).

**Table 3:** Linear regression results from mediation and moderation analyses with level of suicide intent as the outcome^a^.

	**Predictors**	**Outcome**	***B***	**95% CI**	***p***
Model 1	Age	Level of suicidal intent	0.082	0.023, 0.141	0.007^*^
	Female		−0.613	−2.357, 1.130	0.487
	SLEs^b^		0.040	−0.571, 0.652	0.896
Model 2	Age	Social support	−0.004	−0.078, 0.069	0.906
	Female		−0.454	−2.694, 1.787	0.691
	SLEs^b^		−0.785	−1.501, −0.068	0.032^*^
Model 3	Age	Level of suicidal intent	0.082	0.023, 0.141	0.007^*^
	Female		−0.589	−2.334, 1.155	0.505
	SLEs^b^		0.021	−0.591, 0.634	0.945
	Perceived social support		−0.048	−0.145, 0.049	0.329
Model 4	Age	Level of suicidal intent	0.078	0.019, 0.138	0.010^*^
	Female		−0.615	−2.361, 1.132	0.487
	SLEs^b^		0.344	−0.565, 1.253	0.455
	Perceived social support		0.427	−0.567, 1.422	0.396
	SLEs^b^ X perceived social support		−0.043	−0.132, 0.046	0.343

a *Models 1 tests the independent association between SLEs and level of intent. Model 2 tests the independent association between SLEs and perceived social support. Model 3 represents the mediation analysis. Model 4 represents the moderation analysis*.

b *Stressfull life events (SLEs)*.

* *Statistically significant at the 0.05 level*.

### Perceived Social Support as a Moderator of the Association Between Stressful Life Events and Level of Suicide Intent

Perceived social support was not a moderator of the association between stressful life events and level of suicidal intent, as evidenced by a non-significant interaction effect between perceived social support and stressful life events (B −0.043, 95% CI −0.132, 0.046, *p* = 0.343; Table 3).

## Discussion

In this study, we explored the role of perceived social support as a mediator and as a moderator in the relationship between stressful life events and suicidal behavior. We identified that stressful life events were associated with increased risk of suicide attempts and social supported reduced the risk of suicide attempts, however social support did not mitigate the risk of suicide attempts when considered as an effect modifier between stressful events and suicide attempt. We used two outcome variables, the occurrence of suicide attempts and level of suicidal intent, to define suicidal behavior measures, and we did not detect any statistically significant mediation or moderation effects of perceived social support in the association between stressful life events and either of these outcome variables. Our findings are in contrast to a recent study by Yildiz et al. (32) that found perceived social support to mediate the relationship between stressful life events and suicide attempts in a sample of adolescents (32). Our contrasting finding suggests that mediation by perceived social support may not apply to this study sample of adults with suicide attempts admitted to psychiatric hospital, as the cited study was based on a general population sample with mean age of 15 years using different methods of assessing the exposure and outcome measures (32). Alternatively, Yildiz et al. included psychological distress as a partial mediator in the regression model used to assess the role of perceived social support, and the authors state that all variables in the model were imperative to the significance of the mediation (32), which presents the possibility that there may be reason to include a measure of psychological distress in the regression model to adequately assess the mediation effects of perceived social support.

Although there is evidence for moderation by perceived social support (27, 28, 46), and indication in literature that the effect of stress on suicide differs according to differing perceived social support, this was not seen in our study. The studies looking at the moderation effects of perceived social support in the investigated association often included specific populations such as transgender youth (28), women with breast cancer (33), African children diagnosed with HIV (27), and as such, the moderating effect by perceived social support may not be present in our population of psychiatric inpatients with a history of suicidal behavior. Studies conducted in different populations used alternative measures to define perceived social support. For instance, the study on transgender youth used a perceived social support scale that limited responses to friends, significant others, and family (28), whereas the perceived social support measures used in our study do not limit the participant's responses to the type of individual providing the support. Thus, it may be important to evaluate perceived social support from different support figures within the psychiatric population and re-examine their moderating effects.

Despite not detecting mediating or moderating effects of perceived social support, we did find significant associations independently between stressful life events and suicide attempts, and stressful life events and perceived social support. The significance of the independent associations between stressful life events and suicide attempts (12–14), and stressful life events and perceived social support (30, 31), is consistent with what is seen in literature. Our findings lend further support for considering these factors both in statistical models and in clinical practice.

We did not find an association between stressful life events and level of suicidal intent, despite finding an association between stressful life events and suicide attempt. Although a high level of suicide intent is highly predictive of suicidal behavior as seen in literature (47), there is limited literature on the role of perceived social support in the association of stressful life events and level of suicidal intent specifically, which may provide reason as to why an independent relationship between stressful life events and suicidal intent was not established. Furthermore, there is more evidence for the association between stressful life events and severe forms of suicidal behavior such as suicide attempts, as evidenced by a review looking at the association between stressful life events and suicidal behavior in 95 independent studies (29). Thus, when investigating the role of perceived social support in this association, it may be necessary to define the outcome using specific definition of suicidal behavior such as suicide attempt.

## Limitations

This is a cross sectional study limiting the inferences of the associations between stressors and suicide attempts. Furthermore, the stressful life events were reliant on self-report and subject to recall bias. Another limitation of this study is that the perception of social support is relative to the time of recruitment, whereas the participant's suicide attempt predates the time of recruitment and completion of the questionnaires. As such the reported perception of social support at the time of recruitment may have a different relevance on a suicide attempt that has occurred previously. Future directions may consider completing such assessments closer to the time of the suicidal behavior when feasible. An additional limitation regarding the stressful life events is that the scale used for data collection did not have objective weightings associated with the listed events. Thus, it may be important for future research to explore these analyses while also giving weightings to the stressful life events as different life events may have varying levels of impact predates the time of recruitment and completion of the questionnaires relevance Previously. may consider completing such assessments closer to the time of the suicidal behavior when feasible additional not withstanding these limitations, the study provides further data into the role of stressful life events and perceived social support in the association with suicidal behavior in a high-risk group of patients with psychiatric disorders and suicide attempts admitted to psychiatric hospital.

## Conclusion

This study explored the role of perceived social support in the association between stressful life events and suicidal behavior. We found that perceived social support did not act as a mediator or moderator in the investigated association, but there were significant independent relationships between stressful life events and perceived social support and stressful life events and suicide attempts. There is limited evidence on the association between stressful life events and perceived social support, and as such, our study adds to literature by providing evidence for this association in a psychiatric population. The results from this study also reinforce the importance of screening for variables such as stressful events and low perceived social support in the psychiatric population, in order to adequately assess risk for suicidal behavior. Future directions should include the investigation of the role of perceived social support as a mitigating factor in reducing the risk of suicide. Further investigations into the role of perceived social support in outcomes with different aspects of suicidal behavior will also provide clarification on the role of perceived social support in reducing suicidal behavior.

## Data Availability Statement

The raw data and related information supporting the conclusions of this article can be made available upon request.

## Ethics Statement

The studies involving human participants were reviewed and approved by the study was approved by the Hamilton Health Sciences (#10-661) and St. Joseph's Healthcare (#11-3479) Research Ethics Boards. The patients/participants provided their written informed consent to participate in this study.

## Author Contributions

ZS conceived the study and outlined study design and implementation. BP wrote the study manuscript, including the introduction, methods, results, and discussion. TR and MR contributed to the methods section and helped draft the methods and results sections of the manuscript. NS, NB-M, AH, CC, AD'E, LM, LT, and ZS provided feedback and edits for the manuscript. All authors have read and approved this manuscript.

## Funding

This study from which this article has drawn data from was supported by a Brain and Behavior Research Foundation Young Investigator Grant (#19058) awarded to ZS (39). The Brain Behaviour and Research Foundation does not have any role in study design, analysis or reporting of results.

## Conflict of Interest

The authors declare that the research was conducted in the absence of any commercial or financial relationships that could be construed as a potential conflict of interest.

## Publisher's Note

All claims expressed in this article are solely those of the authors and do not necessarily represent those of their affiliated organizations, or those of the publisher, the editors and the reviewers. Any product that may be evaluated in this article, or claim that may be made by its manufacturer, is not guaranteed or endorsed by the publisher.
